# Contribution of HLA class I (A, B, C) and HLA class II (DRB1, DQA1, DQB1) alleles and haplotypes in exploring ethnic origin of central Tunisians

**DOI:** 10.1186/s12920-024-01821-x

**Published:** 2024-02-29

**Authors:** Amène Ben Bnina, Amri Yessine, Yasmine El Bahri, Saoussen Chouchene, Nada Ben Lazrek, Mariem Mimouna, Zeineb Mlika, Aziza Messoudi, Dorsaf Zellama, Wissal Sahtout, Amina Bouatay

**Affiliations:** 1 Hematology Laboratory, Sahloul University Hospital, Sousse, Tunisia; 2 Biochemistry Laboratory, Béchir Hamza Children’s Hospital, Bab Saadoun Square, 1007 Tunis, Tunisia; 3 Hematology Laboratory, Fatouma Bourguiba Teaching Hospital, Sousse, Tunisia; 4 Nephrology Department, Sahloul University Hospital, Sousse, Tunisia; 5 Faculty of Pharmacy, University of Monastir, Monastir, Tunisia; 6 Faculty of Medicine, University of Sousse, Sousse, Tunisia; 7 Department of Educational Sciences, University of Jendouba, Higher Institute of Applied Studies in Humanity Le Kef, Kef, Tunisia

**Keywords:** Human leukocyte Antigen (HLA), Haplotypes, Genetic polymorphism, Anthropology

## Abstract

**Background:**

Estimation of HLA (Human leukocyte Antigen) alleles’ frequencies in populations is essential to explore their ethnic origin. Anthropologic studies of central Tunisian population were rarely reported. Then, in this work, we aimed to explore the origin of central Tunisian population using HLA alleles and haplotypes frequencies.

**Methods:**

HLA class I (A, B, C) and HLA class II (DRB1, DQA1, DQB1) loci genotyping of 272 healthy unrelated organ donors was performed by Polymerase Chain Reaction-Sequence Specific Oligonucleotide (PCR-SSO). We compared central Tunisians with other populations (Arabs, Berbers, Mediterraneans, Europeans, Africans, etc.) using alleles and haplotypes frequencies, genetic distances, Neighbour-Joining dendrogram and correspondence analysis.

**Results:**

Among the 19 HLA A alleles, the 26 HLA B alleles, the 13 HLA C alleles, the 15 HLA DRB1 alleles, the 6 HLA DQA1 alleles and the 5 HLA DQB1 alleles identified in the studied population, HLA A*02 (22.8%), HLA B*50 (13.1%), HLA C*06 (21.8%), HLA DRB1*07 (17.8%), HLA DQA1*01 (32.1%) and HLA DQB1*03 (31.6%) were the most frequent alleles. The extended haplotypes HLA A*02-B*50-C*06-DRB1*07-DQA1*02-DQB1*02 (1.97%) was the most frequent HLA six-loci haplotype.

**Conclusion:**

Central Tunisians were very close to other Tunisian populations, to Iberians and North Africans. They were rather distant from sub-Saharan populations and eastern Mediterraneans especially Arabs although the strong cultural and religious impact of Arabs in this population.

## Introduction

The HLA system is a genetic system located on the short arm of chromosome 6, containing up to 35,000 alleles, registered in the IMGT/HLA database [1, 2]. HLA system codes for surface proteins that initiate the immune response. HLA antigens recognize the foreign peptide, present it to T lymphocytes and thus activate humoral and/or cellular response. However, HLA system is highly polymorphic, enabling the presentation of an enormous repertoire of peptides. This great genetic variability makes this system a genetic predisposition factor for certain diseases, and an obstacle to graft survival in some organ transplantations. Besides, estimation of HLA alleles frequencies in general population is essential in anthropological studies [2–4]. In fact, the high polymorphism of HLA system makes it a valuable tool for classifying populations, exploring their ethnic origins and their immigration patterns. HLA genetic studies have been used in many anthropological studies including African anthropological studies [4–16].

**Fig. 1:**
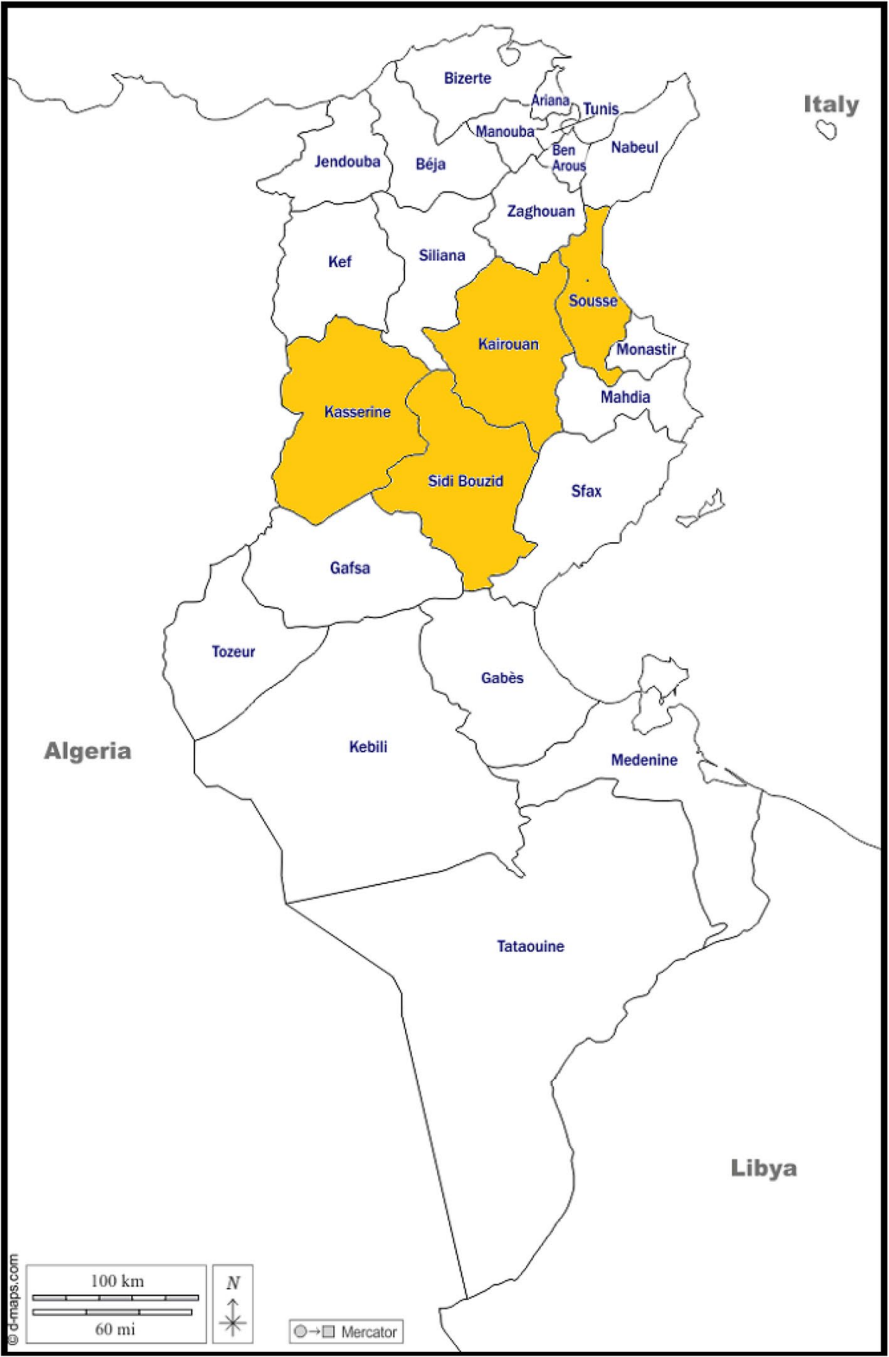
Map showing the central Tunisian governorates included in this study

The main origin of present-day Tunisians is the native Berbers. But the other civilizations that settled in Tunisia also had an impact on the heritage of Tunisians. Indeed, Tunisia, including the central Tunisian region, has seen a succession of several civilizations. It was invaded by the Phoenicians which emigrated from the Middle East to the Mediterranean coast between 1000 and 500 BC. In 815 BC they created Carthage. After that, Romans ruined Carthage in 146 BC and colonized Tunisia. Tunisia was then attacked by the Vandals in the fifth century. Muslims came to Tunisia in the seventh century to found Kairouan; the first Islamic city in North Africa. The Muslim invasion of Tunisia continued between the seventh and fifteenth centuries, with the arrival of Arab tribes from Egypt and the Middle East [17]. Tunisia was then invaded by the Ottoman (Turks) in 1574 AD, followed by French colonization until independence in 1956 [18]. Like all regions of Tunisia, the central region is characterized by great ethnic diversity. Our study focuses on the central Tunisian region especially Sousse, Kairouan, Kasserine and Sidi Bouzid governorates (Fig. 1). Central region of Tunisia comprises nearly 19% of all population according to official census in 2014. Inhabitants of central Tunisia are mainly Arab-speaking populations and Berbers.

The aim of this study was to explore the most likely origin of the population of central Tunisia which was the starting point (especially Kairouan) for the spread of Arabo-muslim civilization in North Africa. This investigation was made using HLA alleles frequencies and comparison with other Tunisian, Mediterranean, sub-Saharan and Arab populations (Table 1).

**Table 1:** Populations included in the present study

Populations	Number of participants	References
Austria	200	[25]
Basques	83	[11]
Belgium	99	[25]
Burkina Faso Mossi	53	[29]
Cantabrians	83	[11]
Central African Republic Aka Pygmy	93	[14]
Central Tunisians	272	Present study
France West Breton	150	[31]
Gaza Palestinians	165	[23]
Germany Essen	174	[25]
Greece Crete	135	[27]
Italy Central	380	[15]
Jordan	15,144	[34]
Kenya	144	[25]
Lebanon pop 2	191	[33]
Libye	118	[32]
Macedonia pop 3	172	[30]
Moroccan_Jews	113	[25]
Morocco Nador Metalsa pop 2	73	[35]
Morocco Settat Chaouya	98	[13]
Portugal North pop 2	1801	[25]
Saudi Arabia pop D	2405	[26]
Spain Granada	280	[28]
Sudan Mixed	200	[24]
Tunisia	100	[8]
Tunisia Gabes	96	[19]
Tunisia Gabes Arab	95	[5]
Tunisia Ghannouch	82	[6]
Tunisia Jerba Berber	55	[5]
Tunisia Matmata Berber	81	[5]
Tunisia pop A	111	[20]
Tunisia pop B	104	[21]
Turkey pop A	250	[25]
Uganda Kampala pop 2	175	[36]
Zimbabwe Harare Shona	230	[22]

## Materials and methods

### Study subjects

Our study included retrospectively 272 healthy unrelated donors from solid organ donors’ database of hematology’s laboratory in Sahloul Hospital of Sousse, Tunisia. All individuals included in the study were donors of solid organs and were from the center of Tunisia namely Sousse governorate, Kairouan governorate, Kasserine governorate and Sidi Bouzid governorate. No ethnic or linguistic criterion was used to include subjects in this study. To participate in the study, informed consent was obtained from all participants. This study was approved by the local ethics committee of Sahloul university Hospital. All data and identities of patients were processed with strict confidentiality.

### Laboratory analysis

#### HLA-A, HLA-B, HLA-C, HLA-DR, and HLA-DQ genotyping

EDTA anticoagulated venous blood samples were used for HLA genotyping. Extraction of genomic DNA from these samples was performed using the “QIAamp DNA mini kit” (Qiagen, Hilden, Germany). Gene typing of the HLA-A, HLA-B, HLA-C, HLA-DR, and HLA-DQ loci was performed by the Polymerase chain reaction-sequence specific oligonucleotide (PCR-SSO) technique with the LABType™ SSO typing kits (One lambda Inc, USA). Interpretation was performed on the HLA FUSION™ software.

## Statistical analysis

In the present study, HLA alleles of the six HLA loci; HLA-A, -B, -C, -DRB1, -DQA1 and -DQB1 frequencies were determined using the gene counting method. Frequencies of haplotypes were estimated through the maximum-likelihood approach from the related genotypic data using the expectation–maximization algorithm [37, 38] via The Arlequin v3.5.2.2 program [39]. To evaluate Hardy–Weinberg equilibrium (HWE) at each locus, a modified Markov-chain random walk approach with 100,172 steps was applied.

Linkage disequilibrium (LD) between specific alleles at distinct loci, along with the significance level (*p* < 0.05) for 2 X 2 comparisons and the relative linkage disequilibrium (RLD or D’), were determined as previously outlined [40]. For phylogenetic tree analysis (dendrograms), the Neighbour-Joining (NJ) method [41] was utilized along with standard genetic distances [42]. The DISPAN software containing GNKDST and TREEVIEW programs [43, 44] facilitated these analyses and the tree construction. Furthermore, a three-dimensional correspondence analysis and its two-dimensional representation were performed with R language using the packages Factoshiny and FactoMineR [45]. This analysis provided an overview of the population relationships in the context of HLA allele frequencies.

## Results

### Alleles frequencies in the studied population

In this study of central Tunisian population, the distribution of HLA genotypes class I and class II were in Hardy Weinberg equilibrium (Table 2). The frequencies of HLA A, B, C, DQA1, DRB1 and DQB1 of Tunisians from the center are illustrated in Table 3. Of the 19 HLA A alleles, A*02 (22.8%), A*01 (13.4%) and A*30 (12.7%) were the most frequent alleles in central Tunisians. They were also the most frequent alleles in Moroccans [35, 46] and in Spanish people [10]. These alleles especially A*01 and A*02 were also in high frequencies in other Tunisian, north African and Iberian populations [6, 7, 11, 12, 16, 21, 47]. As for the 26 HLA B alleles identified, HLA-B*50 (13.1%), HLA-B*44 (9.2%) and HLA-B*51 (9%) were the most frequent alleles. HLA-B*50 and HLA-B*51 are common alleles in Mediterraneans [12, 16, 46]. HLA-B44 and HLA-B50 were also among the most prevalent alleles in the Metalsa Berbers Moroccan population [35]. HLA-B*44 allele was also the most frequent in Tunisian Berbers [9], in the Tunisians-B [21], Spaniards Basques [47] and in high frequencies in Algerians [12], French Basques[48] and in Swiss [49]. Among the 13 HLA C alleles identified, HLA-C*06 (21.8%), HLA-C*07 (18.1%) and HLA-C*04 (13.1%) were the most frequent alleles. In the same way, the three HLA C alleles were the most frequent in North Tunisians [8]. The same result was found in Algerians [12], Moroccans [35] and Spaniards [47].

**Table 2:** Hardy–Weinberg equilibrium and heterozygosity

Locus	Genot	Obs.Het	Exp.Het	P-value	s.d	Steps done
A	272	0.82253	0.87455	0.09254	0.00058	100,172
B	272	0.88816	0.93436	0.13333	0.00044	100,172
C	213	0.77869	0.86710	0.09110	0.00056	100,172
DRB1	174	0.78289	0.87548	0.25414	0.00077	100,172
DQA1	165	0.68421	0.75122	0.08616	0.00064	100,172
DQB1	174	0.71711	0.75688	0.72467	0.00146	100,172

**Table 3:** HLA-A, -B, -DRB1 and -DQB1 allele frequencies

Locus	Allele	Allele Frequency	Locus	Allele	Allele Frequency	Locus	Allele	Allele frequency
**Locus A (2*n* = 544)**	**01**	**13.40**		**51**	**9.00**	**Locus DQB1** **(2*n* = 348)**	**06**	**0.60**
	**02**	**22.80**		**52**	**3.10**		**02**	**29.0**
	**03**	**9.60**		**53**	**2.90**		**03**	**31.60**
	**11**	**1.80**		**57**	**2.40**		**04**	**8.00**
	**23**	**7.90**		**58**	**3.90**		**05**	**11.80**
	**24**	**6.60**		**73**	**0.70**		**06**	**19.50**
	**25**	**0.40**		**78**	**0.20**			
	**26**	**3.50**	**Locus C** **(2n = 426)**	**02**	**5.90**			
	**29**	**5.00**		**03**	**2.60**			
	**30**	**12.70**		**04**	**13.10**			
	**31**	**2.00**		**05**	**5.90**			
	**32**	**3.10**		**06**	**21.80**			
	**33**	**2.60**		**07**	**18.10**			
	**34**	**1.10**		**08**	**3.30**			
	**36**	**0.20**		**12**	**10.60**			
	**66**	**0.60**		**14**	**3.10**			
	**68**	**5.30**		**15**	**6.10**			
	**69**	**0.20**		**16**	**7.50**			
	**74**	**1.30**		**17**	**1.90**			
**Locus B** **(2*n* = 544)**	**07**	**5.80**		**18**	**0.20**			
	**08**	**4.50**	**Locus DRB1** **(2*n* = 348)**	**07**	**17.80**			
	**13**	**3.80**		**04**	**16.10**			
	**14**	**3.90**		**11**	**13.50**			
	**15**	**3.60**		**01**	**5.50**			
	**18**	**4.80**		**03**	**13.50**			
	**27**	**1.50**		**13**	**10.90**			
	**35**	**8.10**		**15**	**10.60**			
	**37**	**1.30**		**08**	**5.20**			
	**38**	**3.30**		**10**	**2.00**			
	**39**	**1.30**		**16**	**1.40**			
	**40**	**2.80**		**12**	**1.10**			
	**41**	**2.20**		**14**	**1.10**			
	**42**	**1.30**		**09**	**0.30**			
	**44**	**9.20**	**Locus DQA1** **(2*n* = 330)**	**01**	**32.10**			
	**45**	**3.30**		**05**	**30.00**			
	**47**	**0.20**		**02**	**17.60**			
	**49**	**4.00**		**03**	**16.40**			
	**50**	**13.10**		**04**	**3.30**			

As regards the HLA class II alleles, 15 HLA DRB1 alleles were identified. Among them, HLA-DRB1*07 (17.8%), HLA-DRB1*04 (16.1%) and HLA-DRB1*03 (13.5%) were the most frequent alleles. HLA-DRB1*07 was also the most frequent allele in Berbers [9] and other Tunisians [6, 16]. HLA-DRB1*03 was also observed in high frequencies in many Mediterranean populations [7, 12, 46, 47]. HLA-DRB1*04 was observed in high frequency in Moroccans [46] and in ghanouchians [6]. Of the 6 HLA DQA1 alleles identified, HLA-DQA1*01 (32.1%) and HLA-DQA1*05 (30%) were the most frequent alleles. These two alleles were also the most frequent HLA DQA1 alleles in Spaniards, Basques [47], in Algerians [12] and in Moroccans [13, 46]. HLA-DQA1*05 and HLA-DQA1*01 were also, in addition of HLA-DQA1*02, the most frequent alleles in North Tunisians[8]. In addition, of the 5 HLA DQB1 alleles identified, HLA-DQB1*03 (31.6%) and HLA-DQB1*02 (29%) were the most frequent alleles. These two alleles were also frequent in many other Mediterranean populations [6, 7, 9, 11, 16, 46, 47].

### Allelic comparison between Tunisians and other populations

Population of central Tunisia were compared with other Mediterranean, Arab-speaking and worldwide populations, using generic HLA-DRB1 and HLA-DQB1 allele frequencies data.

**Fig. 2:**
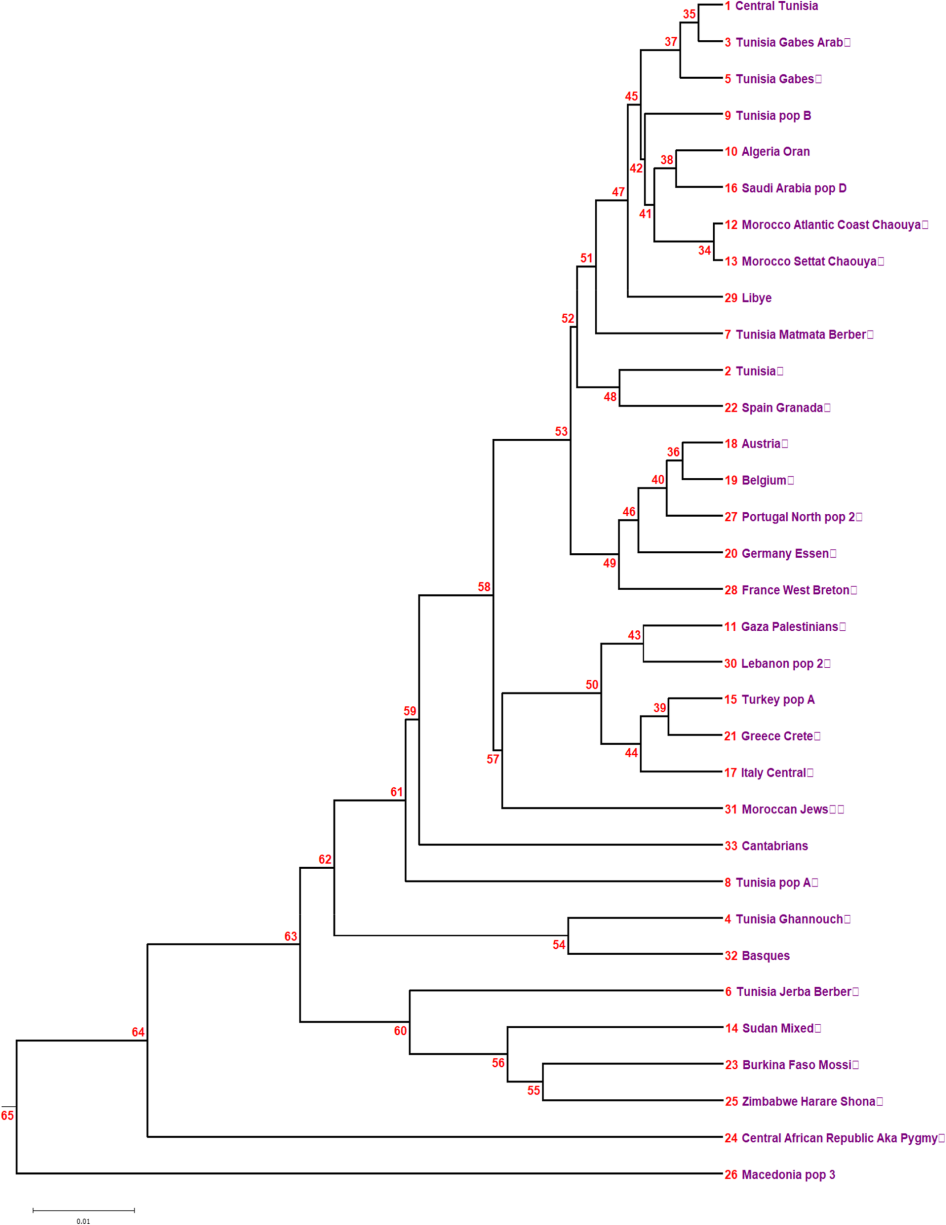
Neighbor-Joining dendrogram showing relatedness between central Tunisians and other populations using generic genotyping of HLA-DQB1 and HLA-DRB1. Standard genetic distances (SGD) between populations were determined. Populations’ data are from references detailed in Table 1. Bootstrap values from 1.000 replicates are shown

**Fig. 3:**
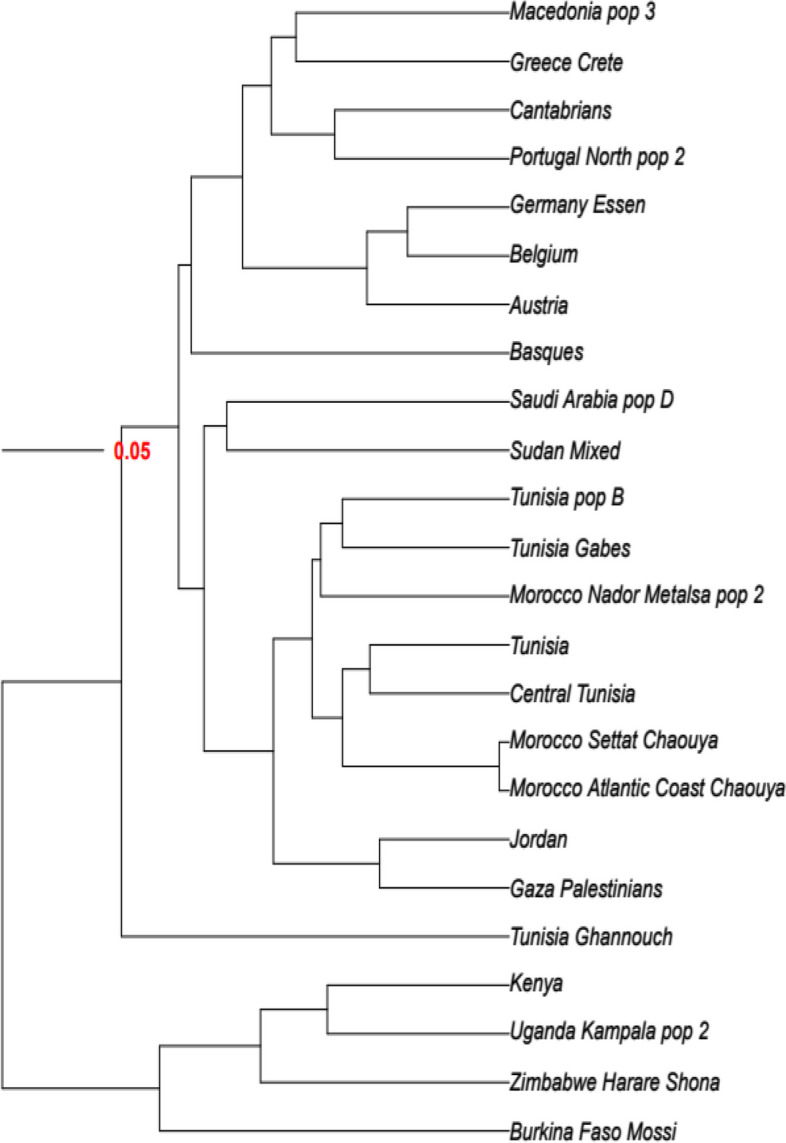
Neighbor-Joining dendrogram showing relatedness between central Tunisians and other populations using generic genotyping of HLA-A and HLA-B. Standard genetic distances (SGD) between populations were determined. Populations’ data are from references detailed in Table 1. Bootstrap values from 1.000 replicates are shown

**Fig. 4:**
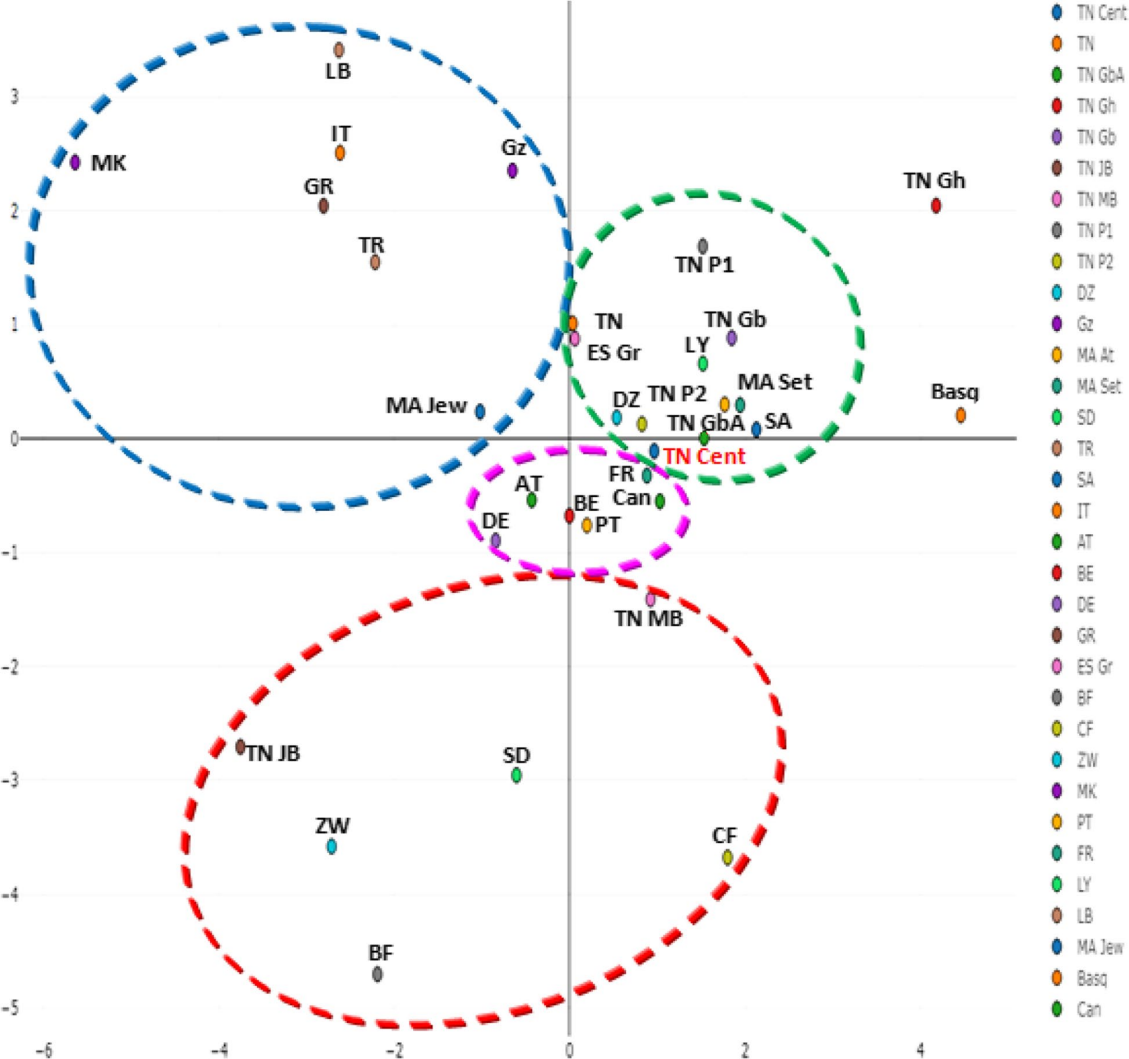
Correspondence analysis (bi-dimensional representation) based on the standard genetic distances, showing a global view of the relationship between central Tunisians and other Mediterranean populations according to HLA DRB1, and HLA DQB1 allele frequencies data

The allelic comparison was done at the levels of Neighbor-Joining (NJ); (Fig. 2 and 3), correspondence analysis (Fig. 4), and standard genetic distances (SGD); (Table 4).

**Table 4:** Standard genetic distances (SGD) between central Tunisians and other populations

Population	Abbreviation	SGD*
Algeria Oran	DZ	0.017
Austria	AT	0.026
Basques	Basq	0.064
Belgium	BE	0.013
Burkina Faso Mossi	BF	0.071
Cantabrians	Can	0.053
Central African Republic Aka Pygmy	CF	0.100
France West Breton	FR	0.031
Gaza Palestinians	Gz	0.026
Germany Essen	DE	0.031
Greece Crete	GR	0.042
Italy Central	IT	0.040
Lebanon pop 2	LB	0.047
Libye	LY	0.025
Macedonia pop 3	MK	0.136
Morocco Atlantic Coast Chaouya	MA At	0.018
Moroccan_Jews	MA Jew	0.050
Morocco Settat Chaouya	MA Set	0.015
Portugal North pop 2	NL	0.011
Saudi Arabia pop D	SA	0.020
Spain Granada	ES Gr	0.029
Sudan Mixed	SD	0.040
Tunisia	TN	0.016
Tunisia Gabes	TN Gb	0.011
Tunisia Gabes Arab	TN GbA	0.005
Tunisia Ghannouch	TN Gh	0.070
Tunisia Jerba Berber	TN JB	0.071
Tunisia Matmata Berber	TN MB	0.019
Tunisia pop A	TN P1	0.055
Tunisia pop B	TN P2	0.015
Turkey pop A	TR	0.031
Zimbabwe Harare Shona	ZW	0.068

^*^SGD based on HLA-DRB1, And DQB1 data

#### NJ dendrogram

We compared generic HLA-DRB1 and HLA-DQB1 alleles’ distribution between central Tunisian population and other populations. Comparison was done with NJ dendrogram based on SGD (Fig. 2). Results from NJ dendrogram show four clusters. The first cluster grouped North Africans (Algerians, Moroccans, Tunisians Berbers and Libyans), Europeans (Spanish, Portuguese, French, Belgians, Germans and Austrians) and Saudi Arabians. The second cluster comprised Eastern Mediterraneans (Palestinians, Lebanese, Turks and Greeks), Moroccan Jews and Italians. The third cluster grouped together some Iberian populations (Cantabrians and basques) and some Tunisian populations (Tunisian population A and Tunisia Ghannouch). The fourth cluster contained Sub-Saharan African populations and Tunisia Jerba Berbers. Macedonians formed an out-group.

We compared also generic HLA-A and HLA-B alleles’ distribution between central Tunisian population and other populations, with NJ dendogram based on SGD (Fig. 3). NJ dendogram shows four clusters. The first cluster grouped Europeans (Cantabrians, Portuguese, French, Belgians, Germans, Austrians and basques), Greeks and Macedonians. The second cluster comprised North Africans (Moroccans and Tunisians), sudanese and Saudi Arab. Eastern Mediterraneans (Palestinians, and Jordanians) form the third cluster. The fourth cluster grouped together some Sub-Saharan African populations and Tunisians from Ghannouch.

#### SGD comparison

Calculation of SGD between central Tunisian population and other populations included in this study was based on generic HLA DRB1 and HLA DQB1 allele frequencies (Table 4). SGD results show that central Tunisians were closer to Western Mediterranean populations than to Eastern Mediterranean populations. In fact, Arab Gabesian Tunisians had the closest genetic distance (5 × 10^−3^) to central Tunisian population followed by Gabesian Tunisians (1.1 × 10^−2^), Portuguese (1.1 × 10^−2^), Belgians (1.3 × 10^−2^), Tunisians-B (1.5 × 10^−2^), Moroccans from Settat Chaouya (1.5 × 10^−2^), Northern Tunisians (1.6 × 10^−2^), Algerians from Oran (1.7 × 10^−2^), Moroccans from Atlantic Coast of Chaouya (1.8 × 10^−2^) and Tunisian Berbers from Matmata (1.9 × 10^−2^). Thus, Tunisians from the center seem to be distant from Eastern Mediterranean populations including Arab populations (Lebanese (4.7 × 10^−2^) and Palestinians (2.6 × 10^−2^)).

#### Correspondence analysis

Correspondence analysis using generic HLA DRB1 and HLA DQB1 shows four clusters (Fig. 4). The first combines Eastern Mediterraneans (Turks, Greeks, Macedonians, Palestinians and Lebanese), Moroccan Jews and Italians. The second grouped together Europeans (Cantabrians, Portuguese, French, Belgians, Austrians and Germans). The third comprised Western Mediterraneans (Algerians, Moroccans, Tunisians, Libyans and Spanish) and Saudi Arabians. The fourth cluster contained Sub-Saharan African populations, Tunisia Jerba Berber and Tunisians from Matmata Berber. Tunisians from Ghannouch and basques form an out-group.

### HLA A, B, DRB1, DQA1 and DQB1 linkage disequilibrium (LD)

The study of central Tunisian HLA haplotypes (Table 5) allows their comparison with other haplotypes found in other populations. Table 5 represents HLA (A, B) and HLA (DRB1, DQB1) two-loci haplotypes with significant LD (*p* < 0.05 in all cases) in central Tunisians. The most frequent HLA two-loci haplotypes in central Tunisians were DRB1*07–DQB1*02 (14.94%), followed by DRB1*03–DQB1*02 (11.78%) and DRB1*11–DQB1*03 (11.78%). DRB1*07–DQB1*02 haplotype was also the most frequent two-loci haplotype in southern Tunisians (18.02%)[7], in Arab Gabes Tunisians (15%), Tunisians from Sousse (17.7%) [16] and in Tunisian Berbers(16.03%)[9]. This two loci haplotype was also present in many other Mediterranean populations at high frequencies namely Moroccans (12.6%) [46] Ghannouchians(16.46%) and Spaniards (17.3%)[47].

**Table 5:** HLA class I (A, B) and class II (DRB1, DQB1) two-loci haplotypes with significant linkage disequilibrium (*P* < 0.05 in all cases) in Central Tunisians

HLA	Haplotype	Haplotype Frequency (HF)	D’	HLA	Haplotype	Haplotype Frequency (HF)	D’
A,B (2*n* = 544)	02 58	0.0165	1.000	DRB1-DQB1 (2*n* = 348)	13 03	0.0402	0.639
	02 51	0.0477	1.000		04 03	0.1063	0.659
	30 13	0.0220	0.977		07 03	0.0287	1.000
	02 18	0.0257	0.976		13 06	0.0603	0.971
	01 07	0.0202	1.000		07 02	0.1494	0.999
	03 50	0.0238	1.000		11 05	0.0114	0.993
	03 35	0.0147	0.527		11 03	0.1178	1.000
	30 50	0.0183	0.706		15 06	0.1034	1.000
	01 57	0.0165	0.809		10 05	0.0201	0.999
	02 49	0.0183	0.618		14 05	0.0114	0.995
	02 50	0.0441	1.000		12 03	0.0114	1.000
	30 44	0.0165	0.689		01 05	0.0545	1.000
	02 44	0.0165	0.683		08 04	0.0201	1.000
	23 44	0.0238	0.668		16 05	0.0143	0.997
	23 50	0.0202	0.926		03 04	0.0114	0.713
DRB1-DQB1 (2*n* = 348)	04 04	0.0448	0.982				
	03 02	0.1178	0.982				
	08 06	0.0258	0.639				

DRB1*03–DQB1*02, known as an Iberian paleo-North African haplotype, was among the most frequent two-loci haplotype in central Tunisian population (11.78%). It was also common in many other west Mediterranean populations. Indeed it was frequent in Tunisian Berbers (11.26%) [9], Tunisians-B (14.08%) [21], Algerians(11.3%)[12], Moroccans (15.3%)[46], Spaniards(13.6%)and Basques (17.5%) [47]. Concerning HLA (A, B) two-loci haplotypes, the most frequent in the population of the Tunisian center were A2-B51 (4.77%) and the A2-B50 haplotype (4.41%). These two-loci haplotypes were also frequent in Berbers, other Tunisian and Mediterranean populations [6, 7, 9, 16, 47].

Concerning HLA (DRB1, DQA1, DQB1) three loci haplotypes, the most frequent were HLA DRB1*07-DQA1*02-DQB1*02 (15.15%) and HLA DRB1*03-DQA1*05-DQB1*02 (12.1%). This was concordant with Tunisian and Mediterranean studies. HLA DRB1*07-DQA1*02-DQB1*02 and HLA DRB1*03-DQA1*05-DQB1*02 were among the most frequent three loci haplotypes in Northern Tunisians [8], Moroccans [46], Algerians [12], Spaniards and Basques [47] (Table 6).

**Table 6:** HLA (DRB1, DQA1, DQB1) three-loci haplotypes with significant linkage disequilibrium (*P* < 0.05 in all cases) in Central Tunisians

HLA	Locus	Frequency	D’
DRB1-DQA1-DQB1 (2*n* = 330)	03 05 02	0.1212	1.000
	04 03 04	0.0424	0.762
	13 05 03	0.0393	1.000
	08 01 06	0.0272	1.000
	04 03 03	0.1090	1.000
	07 02 03	0.0212	0.999
	13 01 06	0.0636	1.000
	07 02 02	0.1515	1.000
	11 01 05	0.0121	1.000
	11 05 03	0.1060	1.000
	15 01 06	0.1060	1.000
	14 01 05	0.0121	0.998
	01 01 05	0.0515	0.999
	08 04 04	0.0212	1.000
	16 01 05	0.0121	1.000
	03 04 04	0.0121	1.000

### HLA class I and class II extended haplotype analysis

Extended A-B-DRB1-DQB1 haplotypes and their frequencies in central Tunisian population are represented in Table 7. The most frequent A-B-DRB1-DQB1 extended haplotype in the population of the center of Tunisia was A*02-B*50-DRB1*07-DQB1*02 (3.94%) followed by A*02-B*51-DRB1*13-DQB1-06 (1.97%). HLA A*02-B*50-DRB1*07-DQB1*02 haplotype was also the most frequent haplotype in Tunisians (from the North and the south of Tunisia) (2.2%) [16, 50], in Tunisian Berbers (8.1%) [9], Southern Tunisians(3.2%)[7], in Gabesians(2.6%)[19], in Ghanouchians (1.8%) [6], in Tunisians-B (1.2%) [21], in Spaniards (1.2%) [47], in Turks (1.3%) [51] and Moroccan Jews (2%) [25]. The second most frequent four loci-haplotype A*02-B*51-DRB1*13-DQB1*06 (1.97%), another Ibero-berber haplotype [46], was also frequent in Moroccans [46],and Spaniards [10].

**Table 7:** The most frequent four-loci haplotype in Central Tunisians

HLA	Locus	Frequency	D’
A-B-DRB1-DQB1 (2*n* = 304)	02 51 13 06	0.0197	0.955
	03 50 04 04	0.0131	0.724
	02 50 07 02	0.0394	0.946
	02 51 07 02	0.0164	0.816
	02 07 15 06	0.0131	0.771
	23 44 07 02	0.0131	0.783

The most frequent extended six-Loci haplotypes (A, B, C, DRB1, DQA1, DQB1) and their frequencies are represented in Table 8. The extended haplotypes HLA A*02 -B*50- C*06-DRB1*07-DQA1*02-DQB1*02 (1.97%) and HLA A*02-B*51-C*16-DRB1*07-DQA1*02-DQB1*02 (1.64%) were the most frequent HLA six-loci haplotypes. The first haplotype was also the most frequent in Moroccans from Chouaya[13] and among the most frequent HLA six-loci haplotypes in Northern Tunisians[8]. However, the second extended haplotype HLA A*02-B*51-C*16-DRB1*07-DQA1*02-DQB1*02 was absent in Iberian, Mediterranean and Tunisian populations [6–8, 11–13, 32, 46].

**Table 8:** The most frequent six-loci haplotype in Central Tunisians

HLA	Locus	Frequency	D’
A-B-C-DRB1-DQA1-DQB1 (2*n* = 304)”	26 38 12 13 01 06	0.0098	0.802
	03 52 12 07 02 02	0.0098	0.586
	01 08 07 03 05 02	0.0098	0.961
	03 50 06 04 03 04	0.0098	0.742
	02 50 06 07 02 02	0.0197	0.993
	30 13 06 07 02 02	0.0098	0.999
	01 58 07 13 05 03	0.0098	0.575
	23 50 06 07 02 02	0.0131	0.970
	23 50 06 11 05 03	0.0098	1.000
	30 38 12 13 01 06	0.0098	1.000
	02 18 07 04 03 03	0.0098	0.996
	24 07 07 15 01 06	0.0098	1.000
	02 07 07 15 01 06	0.0098	0.722
	02 51 16 07 02 02	0.0164	1.000
	02 50 06 16 01 05	0.0098	0.951
	34 08 07 03 05 02	0.0098	0.531

## Discussion

To the best of our knowledge, this is the first anthropologic published study of the population of the central Tunisian region in terms of HLA alleles and haplotypes, and the relationship of this population to other populations. For this study, we did not only use allele and haplotype frequencies, but also we used methods of anthropological and evolutionary analysis (genetic distance, correspondence analysis, dendrograms, etc.).

### Central Tunisians, other Tunisians and North Africans

The study of genetic distances and allelic frequencies has shown that the central Tunisian population is closely related to the southern Tunisians, especially the population of Gabes [5, 19], and to northern Tunisians [8, 21]. This shows that central Tunisians are not distinct from other Tunisians. Indeed, all regions share almost the same historical events. However, the population of Jerba Island was genetically rather distant from the central Tunisian region. Moreover, this genetic distance from the Jerba region was also found in other North Africans. This could be explained by the low level of exogamy within the Jerba Berber population (less than 6%) [5]. Besides, according to Khodjet el khil et al., the Jerba population is rather close to some European populations [52]. Then, central Tunisians are closely related to most of Tunisians. Nevertheless, we have found in our population an extended haplotype HLA A*02-B*51-C*16-DRB1*07-DQA1*02-DQB1*02 which was absent in other Tunisian and Mediterranean populations [6–8, 11–13, 32, 46]. Thus, this haplotype could be a characteristic of the population of central Tunisia and reflects the important mixing of populations in this region.

Furthermore, central Tunisians were much closer to western Mediterraneans, particularly North Africans, than to the eastern Mediterraneans. This was confirmed by NJ dendrogram, corresponding analysis, allele frequencies and haplotypes frequencies. These results were also found in the other Tunisian populations [5, 7, 8, 19, 21]. The relatedness found in our study between the population of central Tunisia and North African populations especially Algeria and Morocco can be explained by almost similar historical events. Indeed, the original inhabitants of North Africa (including Tunisians) were the Berbers. These countries were then colonized by the Phoenicians around 1000 BC, then the Romans after Punic wars (264–266 BC). Later, there were Arabs and Muslims conquest in seventh century AD, and important Bedouin immigration in the eleventh century. Then, there was immigration of Andalusians and Negroid slaves. This similar succession of civilizations in each of the countries of North Africa explains the links of relatedness between these different countries [50, 53, 54].

### Central Tunisians and Iberians

NJ dendrogram, genetic distances, corresponding analysis, alleles and haplotypes comparison have shown that the closest populations to central Tunisians were Western Mediterranean populations especially North Africans and Iberians. This result was also found in most of Tunisian regions [5, 7, 8, 16, 19, 21, 50]. The relatedness between central Tunisians, North Africans and Iberians is explained by many historical events. In fact, North African Berbers (including Tunisians and Central Tunisians) were forced to immigrate to the northern coast of the Mediterranean (Spain, Portugal, etc.) due to hyper-arid climatic conditions. This occurred likely in 10,000–4000 BC [27]. On the other hand, North Africans and Iberians were colonized by nearly the same civilizations. Both were invaded by Phoenicians, Romans, Germans, Arabs and Muslims. Besides, Muslim invasion of the Iberians was launched mainly from North Africa and led by North African Muslims and Berbers. The latter settled in Spain specifically for 8 centuries [7, 55]. These events and observations confirm the gene flow and admixture between Iberians and North Africans (including Central Tunisians) and support their relatedness.

### Central Tunisians, Berbers and Arabs

This genetic study of HLA system has revealed that central Tunisians, Algerians, Moroccans, North Tunisians and Southern Tunisians are related to Berbers. This result is not surprising. It is consistent with geography and ancestry. Then, Tunisians and North Africans of the present days are not genetically different from Berbers [9, 16]. However, the genetic impact of Eastern Mediterranean populations, particularly Arabs, on the population of central Tunisia was not significant. Indeed, this study showed that the central Tunisian population is closer to western Mediterranean populations than to eastern ones. Our population was distant from Arabs especially Lebanese and Palestinians. This could be explained by the low influx of Middle East Arabs compared with the settled Berbers [7]. This low genetic contribution of Arabs in Tunisians can be also explained by the low level of mixture between Berber tribes and Arab tribes [9]. In fact, during invasion of Tunisia by the two Arab Tribes (Beni Hilal and Beni Souleim), Berbers were obliged to take refuge in mountains for fear of persecution by the invaders [7, 9, 50]. In addition, the barriers of language, religion and traditions between Arabs and Berbers were additional factors in this low admixture. Nevertheless, although genetic flow from Arabs was low in Tunisia and North Africa, social and cultural effect was very significant evidenced by the adoption of Arab speaking language and Islam religion in all of North African countries. However, Saudi Population was also genetically quite close to central region of Tunisia. This can be probably explained by the by Arab immigration during the Islamic conquests.

### Central Tunisians, Blacks and sub-Saharans

Tunisian Blacks are more present in southern Tunisia than in northern Tunisia. Their origins lie mainly in the area extending from Lake of Chad to West Africa [7]. The second origin of Tunisian Blacks was Arab invasion of North African region. However, our genetic analysis of the HLA system has shown that central Tunisians are distant from sub-Saharan populations. This is consistent with the results of other studies which have found that Tunisians, even southern Tunisians, were genetically distant from sub-Saharan Africans [6, 7, 9, 16, 50]. This is probably due to the high endogamy within these black populations, where inter-ethnic marriages were rare because of cultural barriers. This has reduced admixture between sub-Saharan populations and other Tunisians, and limited their genetic contribution to the Tunisian genetic pool [7].

This study does have its limitations, namely the generic typing of HLA alleles, given the lack of resources. However, the strengths of this study are the typing of the 6 HLA-A, -B, -C, -DRB1, -DQA1, DQB1 loci. Indeed, most anthropological studies based on the HLA system have not studied the HLA-C and HLA-DQA1 loci. Moreover, to our knowledge, this is the first anthropological study of the population of the Tunisian center.

In conclusion, according to this study, central Tunisians were very close to other Tunisian populations, including the Berbers. They were also close to North Africans and Iberians. However, central Tunisians were genetically rather distant from eastern Mediterranean populations and sub-Saharans, especially Arabs. This result is somewhat surprising, although it is consistent with other Tunisian studies. Indeed, despite the various Arab invasions of Tunisia and the strong cultural and religious impact, there was no significant Arab contribution to the Tunisian gene pool.

## Data Availability

For Data evaluation please contact Dr. Ben Bnina Amène at; amenebenbnina@gmail.com.
